# TC2N: A Novel Vital Oncogene or Tumor Suppressor Gene In Cancers

**DOI:** 10.3389/fimmu.2021.764749

**Published:** 2021-12-02

**Authors:** Hanyang Li, He Fang, Li Chang, Shuang Qiu, Xiaojun Ren, Lidong Cao, Jinda Bian, Zhenxiao Wang, Yi Guo, Jiayin Lv, Zhihui Sun, Tiejun Wang, Bingjin Li

**Affiliations:** ^1^ Department of Radiotherapy, The Second Hospital of Jilin University, Changchun, China; ^2^ Department of Thyroid Surgery, The Second Hospital of Jilin University, Changchun, China; ^3^ Department of Hepatobiliary and Pancreatic Surgery, The Second Hospital of Jilin University, Changchun, China; ^4^ Department of Pathology, The Second Hospital of Jilin University, Changchun, China; ^5^ Department of Biobank, The China-Japan Union Hospital of Jilin University, Changchun, China; ^6^ Department of Breast Surgery, The Affiliated Hospital Changchun University of Chinese Medicine, Changchun, China; ^7^ Department of Orthopedics, The China-Japan Union Hospital of Jilin University, Changchun, China; ^8^ Department of Pharmacy, The Second Hospital of Jilin University, Changchun, China; ^9^ Jilin Provincial Key Laboratory on Molecular and Chemical Genetic, The Second Hospital of Jilin University, Changchun, China

**Keywords:** TC2N, tumor-associated antigens (TAAs), cancer, signal pathway, molecular mechanism, functional characterization, clinical feature

## Abstract

Several C2 domain-containing proteins play key roles in tumorigenesis, signal transduction, and mediating protein–protein interactions. Tandem C2 domains nuclear protein (TC2N) is a tandem C2 domain-containing protein that is differentially expressed in several types of cancers and is closely associated with tumorigenesis and tumor progression. Notably, *TC2N* has been identified as an oncogene in lung and gastric cancer but as a tumor suppressor gene in breast cancer. Recently, a large number of tumor-associated antigens (TAAs), such as heat shock proteins, alpha-fetoprotein, and carcinoembryonic antigen, have been identified in a variety of malignant tumors. Differences in the expression levels of TAAs between cancer cells and normal cells have led to these antigens being investigated as diagnostic and prognostic biomarkers and as novel targets in cancer treatment. In this review, we summarize the clinical characteristics of TC2N-positive cancers and potential mechanisms of action of TC2N in the occurrence and development of specific cancers. This article provides an exploration of TC2N as a potential target for the diagnosis and treatment of different types of cancers.

## 1 Introduction

Cancer is an important public health concern worldwide and continues to be of great interest to the scientific community. It is one of the leading causes of death, with approximately 14 million new cases and 8.2 million cancer-related deaths occurring in 2018 (1). This disease is considered the biggest barrier to improving life expectancy in countries in the 21st century (2). Annually, over 4 million new cancer patients and over 2 million cancer-related mortalities are reported in China. Despite the availability of multiple treatment modalities such as surgery, chemotherapy, radiation therapy, and targeted therapy, the 3- and 5-year cancer-specific survival rates remain poor (3–7). While overall cancer related mortalities have decreased (8), it is notable that the reduction in mortality is largely due to early detection and prevention rather than development of better treatment options (9–12).

Most cancers are asymptomatic in the early stages of development (13, 14) largely because of their ability to evade immune surveillance (15, 16). Immune evasion is thought to be driven by two major mechanisms. First, owing to altered antigen presentation or receptor library editing, the immune system is unable to detect tumor populations. Second, the initially effective immune response may become ineffective owing to the presence of an immunosuppressive tumor microenvironment (17–19). Therefore, it is important to explore mechanisms of cancer development to identify new markers for diagnosis and prognosis and to develop effective and novel treatment methods. Developments in both fronts will have substantial implications for improving survival rates of cancer patients.

Numerous studies have shown that certain genes, such as oncogenes and tumor suppressor genes, are risk factors for many types of cancer (20–24). When activated, oncogenes stimulate tumor growth whereas tumor suppressor genes prevent tumor growth and development. In mouse models, where oncogene expression is driven by tissue-specific promoters, tumors appear at high frequency but disappear when the inductive stimulus is turned off (25–27), suggesting that oncogenes are the Achilles’ heel of cancer (28). Tumor suppressor genes play a critical role in controlling the cell cycle assuring proper proliferation and differentiation (29). Therefore, identifying these genes is crucial because targeting them may prevent or treat different types of cancers.

The C2 domain was initially thought to be a protein structural domain of calcium-dependent protein kinase C (30–32). Further studies confirmed that the function of the C2 domain was not only calcium-dependent phospholipid binding, but also involved in cellular signal transduction and protein-protein interactions (33). Several proteins that contain a structural domain called the C2 domain have been linked to the regulation of tumorigenesis. For example, a C2 domain-containing protein, DOC2B, plays a tumor-suppressive role in cervical cancer by inhibiting cell proliferation, migration, and invasion (34). Conversely, another C2 domain-containing protein, myoferlin, plays a tumor-enhancing role by promoting metastasis in patients with triple-negative breast cancer (35). Gene encoding tandem C2 domains nuclear protein (TC2N)—a putative C2 domain-containing protein—has recently been shown to function both as an oncogene and a tumor suppressor gene (36–38). TC2N is located on human chromosome 14q32, belongs to the carboxyl-terminal type (C-type) tandem C2 protein family, and contains two C-terminal C2 domains (C2A and C2B) (39). *TC2N* is also an immune system gene similar to *IFI27*, *CASS4*, and *SMARCD3* (40). Given its tumorigenesis properties and its association with the immune system, it has been proposed as a potential target for the detection and treatment of various cancers. In this review, we summarize recent progress in understanding the role and underlying mechanisms of *TC2N* in the occurrence and development of cancer, with a focus on lung cancer, breast cancer, and gastric cancer.

## 2 TC2N in Cancers

*TC2N* expression is upregulated in different types of cancers, including lung, breast, and gastric cancers. The relevant clinicopathological features and the molecular mechanisms of *TC2N* in these cancers are summarized in **Table 1** and detailed in the rest of this section.

**Table 1:** Functional characteristics and clinical features of *TC2N* in human cancers.

Cancer types	Expression	Role	Functional role	Related genes	Clinical features	References
Lung cancer	Upregulated	Oncogene	Promotes proliferation, migration, and invasion and inhibits apoptosis	*CDK5, P53, P21, BAX, BCL1, IκB, NF-κB, MMP7, MMP9*	Advanced TNM stage, high histological grade, and poor clinical prognosis	(36, 41)
Breast cancer	Upregulated	Anti-oncogene	Inhibits proliferative and colony-forming abilities	*ALK, EBP1, P55γ, AKT, Caspase-3, GSK3β, MYC, BAD, PTEN*	Early clinical stage, small tumor size, low lymph node metastasis, high HER-2 positive rate, and good prognosis	(37)
Gastric cancer	Upregulated	Oncogene	Promotes proliferation, migration, and invasion	*MMP2, MMP9, CATSPERB, GALNT3, RBM47*	Advanced TNM stage, large tumor size, high histological grade, advanced distant metastasis, and poor prognosis	(38, 42)

TC2N, tandem C2 domains nuclear protein; CDK5, cyclin-dependent kinase 5; P21 (CDKN1A), cyclin dependent kinase inhibitor 1A; P53, tumor protein p53; BAX, a member of the B-cell lymphoma-2 (BCL2) gene family; BCL1, B-cell lymphoma-1; IκB, inhibitor of NF-κB; NF-κB, nuclear factor kappa-light-chain-enhancer of activated B cells; MMP7, matrix metalloproteinase 7; MMP9, matrix metalloproteinase 9; ALK, anaplastic lymphoma kinase; EBP1, ErbB-3 binding protein 1; AKT, serine threonine kinase; GSK3β, glycogen synthase kinase-3β; BAD, Bcl-2-associated death promoter; PTEN, phosphatase and tensin homolog deleted on chromosome 10; MMP2, matrix metalloproteinase 2; GALNT3, polypeptide N-acetylgalactosaminyltransferase 3; RBM47, RNA binding motif protein 47.

### 2.1 Lung Cancer

#### 2.1.1 Functional Characteristics and Clinical Features of TC2N in Lung Cancer

TC2N is overexpressed in cancerous lung tissues and cell lines compared with that in normal lung tissues and a human bronchial epithelial cell line, respectively. Hao XL et al. (36) showed that upregulation of TC2N was significantly correlated with advanced TNM stage and high histological grade of disease. Additionally, high TC2N expression levels were associated with poor clinical outcomes and significantly short overall survival. Hence, TC2N expression has been proposed as an independent prognostic factor affecting patient survival. Mechanistically, overexpression of TC2N significantly inhibited apoptosis, promoted cell proliferation, and increased migration and invasion of tumor cells *in vitro*; in contrast, knockdown of TC2N promoted apoptosis and inhibited proliferation of lung cancer cells (41). Furthermore, knockdown of TC2N in tumor tissues resulted in an increase in apoptotic cells, supporting the hypothesis that TC2N overexpression promotes tumorigenesis and growth of lung cancer tumors *in vivo*.

In summary, *TC2N* is a potential novel oncogene in lung cancer, whose expression levels are correlated with cancer progression and patient survival. *TC2N* stimulates cell proliferation, migration, and invasion and reduces apoptosis of lung cancer cells *in vitro* and *in vivo*.

#### 2.1.2 Signaling Pathways Influenced by TC2N in Lung Cancer

##### 2.1.2.1 TC2N Inhibits p53 Signaling Pathway in Lung Cancer

**Figure 1:**
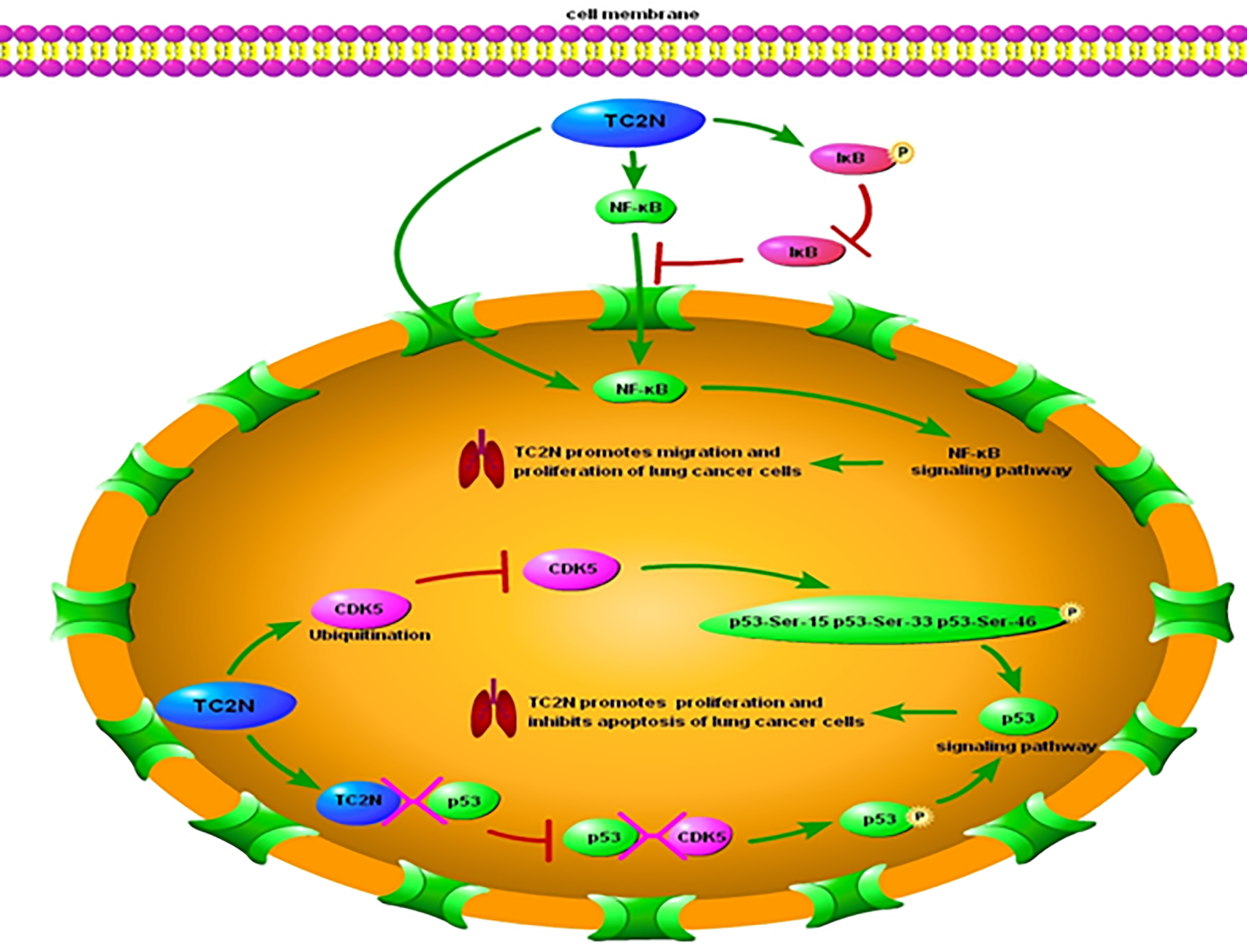
Underlying molecular mechanisms of TC2N in p53 and NF-κB signaling pathways in lung cancer. TC2N, tandem C2 domain nuclear protein; CDK5, cyclin-dependent kinase 5; P53, tumor protein p53; IκB, inhibitor of NF-κB; NF-κB, nuclear factor kappa-light-chain-enhancer of activated B cells.

Hao XL et al. (36) proposed that the regulation of cell proliferation, cell cycle, and apoptosis by TC2N is dependent on the p53 signaling pathway. *TP53*, which encodes p53, was initially classified as an oncogene due to its ability to transform cells (43–46). However, the identification of growth-inhibiting and temperature-sensitive mutants of p53 in sporadic cancer samples and familial cancers has shown that p53 is in fact a tumor suppressor protein (47–53). p53 functions as the major regulator of central signaling and cell fate decision pathways (54). It is a nuclear transcription factor composed of 393 amino acids with four major functional domains: a transcriptional, a DNA binding, a tetramerization, and a regulatory domain (55). It modifies the expression of multiple genes involved in a variety of biological processes, including cell cycle, apoptosis, senescence, differentiation, and DNA repair (56–66). Moreover, p53 has been associated with diverse biological processes, such as regeneration (67), metabolism (68, 69), interaction with viruses (70), prevention of liver pathologies (71, 72), forming a barrier to stem cell formation (73, 74), endocrinology circuits (75) and serving as the guardian of the tissue hierarchy (76). p53 activity is largely controlled by post-translational modifications, such as phosphorylation (77). CDK5 is a protein kinase that phosphorylates p53 at Ser-15, Ser-33 and Ser-46 (78, 79) and binding of CDK5 to p53 induces activation of the p53 signaling pathway (77). Overexpression of TC2N interferes with CDK5-p53 interaction in the nucleus and induces significant CDK5 degradation by increasing the ubiquitination of CDK5 (**Figure 1**). Therefore, an increase in TC2N levels suppresses CDK5-induced p53 phosphorylation and p53 pathway activation. The expression of other key players in the p53 signaling pathway, such as P21, BAX, and BCL-2, is also downregulated by TCN2 (36). When cells experience stress or undergo uncontrolled division and proliferation, p53 is activated (56, 80). Under these conditions, p53 induces p21 expression, causing cell cycle arrest (81, 82). Furthermore, p53 triggers programmed cell death by triggering apoptosis-related genes, including *bax*, a pro-apoptotic member of the bcl-2 family, when a DNA damage cannot be repaired (83). Hence, reduction of the downstream players in the p53 pathway by TCN2 promotes proliferation and prevents apoptosis.

##### 2.1.2.2 TC2N Promotes NF-κB Signaling Pathway in Lung Cancer

In addition to suppressing the p53 pathway, TCN2 was observed to affect another key signaling pathway in lung cancer cells (41). Over 30 years ago, Sen et al. (84) identified a protein that bound to a specific, conserved DNA sequence in the nucleus of activated B lymphocytes. The protein was named after the identified cell type and the gene it affected: nuclear factor binding near the k light-chain gene in B cells (NF-kB) (85). Since its discovery, NF-κB has been found to be involved in several key processes such as immune regulation, inflammation, cell survival, stress response, embryogenesis, differentiation, proliferation, and cell death (86–96) and it functions primarily by orchestrating the expression of many functionally diverse genes (85, 89, 93, 97, 98). Due to its extensive physiological effects, dysregulation of NF-κB can lead to severe consequences (99, 100), including cancer, neurodegenerative diseases, autoimmune diseases, cardiovascular diseases, and diabetes (85, 99–105).

Most lymphatic or solid tumors, including lung cancer, present with increased NF-κB levels (106). NF-κB in the nucleus is an indicator of active NF-κB signaling, and its levels correlate with the transcription of its target genes (107). Typically, NF-κB levels in the nucleus and its activity are regulated by inhibitor of NF-κB (IκB). IκB acts as a gatekeeper, limiting NF-κB migration into the nucleus by masking its nuclear localization domains (108, 109). Additionally, it prevents activation of NF-κB target genes by masking the DNA-binding domains of NF-κB (108), thereby leading to interruption of the NF-κB signaling pathway. Notably, overexpression of NF-κB—both in the nucleus and cytoplasm of lung cancer cells—correlated with increased expression level of TC2N in these cells (41). Hao XL et al. (41) proposed that this increase in NF-κB expression level is a direct consequence of TC2N overexpression in these cells (**Figure 1**). Overexpression of TC2N enhanced the phosphorylation of IκB but decreased the total IκB protein levels, leading to increased nuclear translocation of NF-κB and subsequent activation of the signaling pathway (41). Additionally, TC2N modulates this process through other downstream proteins in the pathway such as MMP7 and MMP9 (41).

### 2.2 Breast Cancer

#### 2.2.1 Functional Characteristics and Clinical Features of TC2N in Breast Cancer

Similar to that in lung cancer tissues, the expression of TC2N was markedly upregulated in breast cancer tissues compared with that in adjacent non-cancerous tissues (37). However, unlike that in lung cancer, upregulated TC2N was associated with good prognosis and overall survival. It positively correlated with the early clinical stage of disease, small tumor size, low lymph node metastasis, and high human epidermal growth factor receptor 2 (HER-2) positivity rate. Additionally, upregulated TC2N inhibited the proliferative and colony-forming abilities of breast cells both *in vitro* and *in vivo*. In summary, in contrast to its role in lung cancer cells, TC2N is a potential tumor suppressor in breast cancer.

#### 2.2.2 Signaling Pathways Influenced by TC2N in Breast Cancer

##### 2.2.2.1 TC2N Inhibits PI3K/AKT Signaling Pathway in Breast Cancer

To explain the tumor suppressive function of TC2N in breast cancer cells, Hao XL et al. (37) proposed that upregulation of TC2N represses the Phosphoinositide 3-kinases/serine-threonine kinase (PI3K/AKT) signaling pathway, which is typically constitutively active in some human cancers (110, 111). PI3K/AKT is a growth-regulating cellular pathway and it is well established that PI3K/AKT signaling enhances tumor cell survival, proliferation, and motility in different tumor types (112–119). PI3Ks form a family of kinases that are expressed in almost all mammalian cells and play essential roles in survival, migration, cell cycle progression, and cell growth (120). PI3K phosphorylates phosphatidylinositol to form inositol lipid, which functions as a second messenger in the human body (121). Similarly, AKT is involved in various physiological processes and is a key regulatory protein for cell growth, survival, metabolism, and proliferation (116, 122–124). The pathogenesis of a variety of human cancers is associated with aberrant regulation of the PI3K/AKT pathway (125–127).

**Figure 2:**
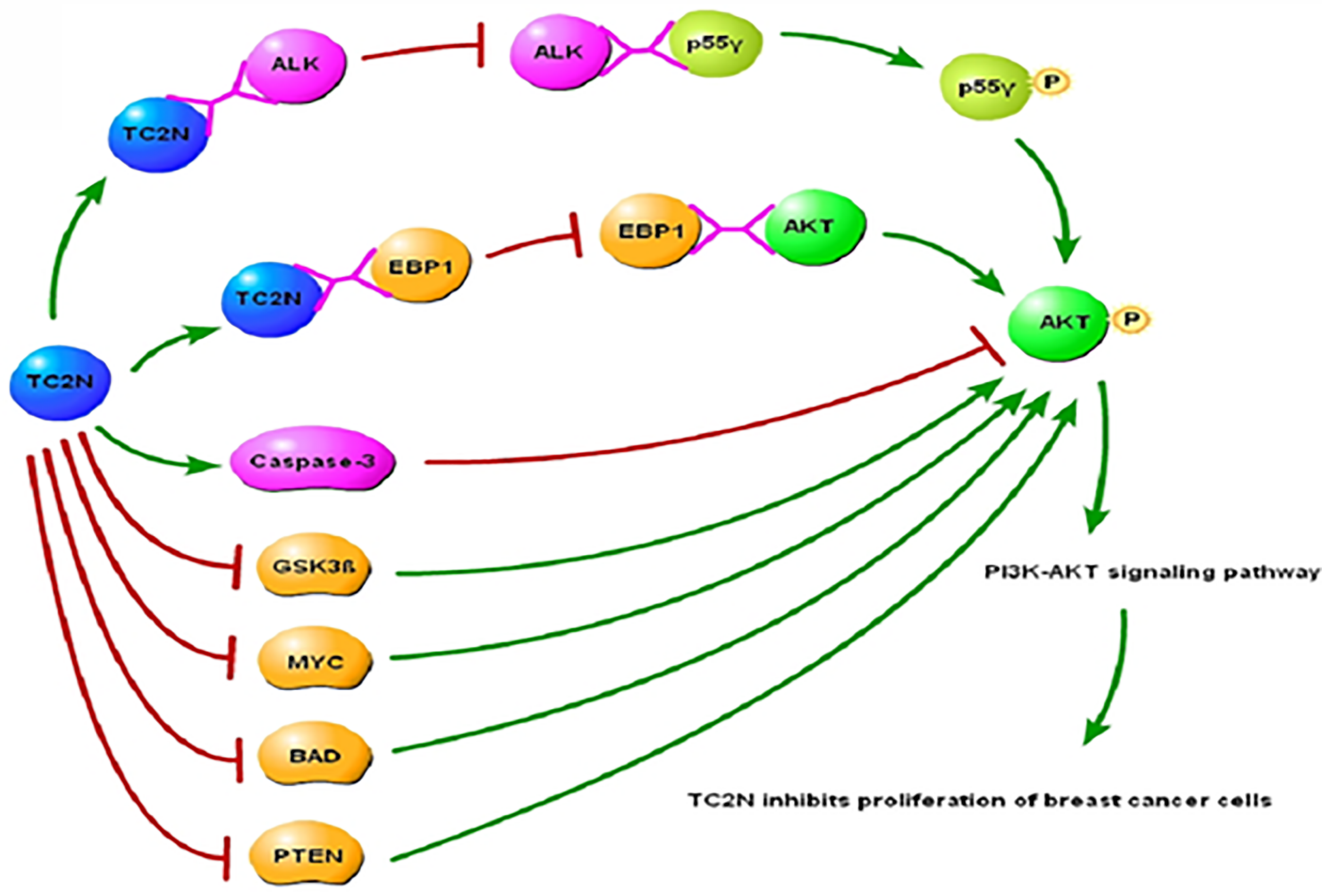
Underlying molecular mechanisms of TC2N in PI3K-AKT signaling pathway in breast cancer. TC2N, tandem C2 domain nuclear protein; ALK, anaplastic lymphoma kinase; EBP1, ErbB-3 binding protein 1; AKT, serine threonine kinase; GSK3β, glycogen synthase kinase-3β; BAD, Bcl-2-associated death promoter; PTEN, phosphatase and tensin homolog deleted on chromosome 10.

Anaplastic Lymphoma Kinase (ALK) is an activator of the PI3K/AKT signaling pathway, and it induces phosphorylation of the p55γ subunit of PI3K in cancer cells, rather than the usual p85 subunit that is phosphorylated (128, 129). It has been shown that the interaction between ALK and p55γ is crucial for ALK-induced p55γ phosphorylation (128) and subsequent activation of the PI3K/AKT signaling pathway. Another key regulatory step in the activation of PI3K/AKT signaling is phosphorylation of AKT by ErbB-3 binding protein 1 (EBP1). TC2N targets both these key steps to inhibit the PI3K/AKT signaling pathway. TC2N forms a complex with ALK, which prevents the ALK-p55γ interaction and therefore inhibits downstream AKT phosphorylation and consequently the PI3K-AKT signaling pathway (**Figure 2**). Additionally, TC2N inhibits the interaction of EBP1 with AKT, which is necessary for phosphorylation of AKT (130, 131) and subsequent PI3K/AKT signaling. Upregulation of TC2N has also been shown to activate AKT inhibitors such as caspase-3 and block AKT inhibitors such as GSK3β, MYC, BAD, and PTEN.

### 2.3 Gastric Cancer

#### 2.3.1 Functional Characteristics and Clinical Features of TC2N in Gastric Cancer

Similar to lung and breast cancer cells, TC2N is overexpressed in a variety of gastric cancer cell lines and tumor samples compared to normal cells and tissues (38, 42). High TC2N levels were significantly correlated with poorly differentiated histological classification, large tumor size, advanced TNM stage, and advanced distant metastasis. Furthermore, patients with high TC2N expression showed poorer prognosis regardless of TNM stage compared to patients with low TC2N expression. *In vitro*, TC2N knockdown significantly inhibited the proliferation of gastric cancer cells, while TC2N overexpression promoted the growth of these cells. Similar results were observed *in vivo* where downregulation of TC2N inhibited the migration and invasion of gastric cancer cells, whereas overexpression had the opposite effect. Thus, similar to lung cancer, *TC2N* potentially functions as an oncogene in gastric cancer.

#### 2.3.2 Signaling Pathway of TC2N in Gastric Cancer

The mechanism of action of TC2N in gastric cancer remains unclear. Shen L et al. (42) suggested that TC2N might partially affect the migration and invasion ability of gastric cancer by regulating the expression levels of MMP2 and MMP9. MMP2 and MMP9 are known to be involved in cell invasion and tumor metastasis (132). TC2N expression also showed strong positive correlation with the expression of CATSPERB and other cancer related genes such as *GALNT3* and *RBM47* (38). However, the detailed molecular mechanism by which TC2N promotes gastric cancer progression needs further evaluation.

## 3 Conclusion and Future Perspectives

High-throughput gene expression profiling facilitates the simultaneous measurement of the expression levels of thousands of genes. A key application of gene expression profiling in cancer is to identify differences in gene expression patterns between tumor and control samples (133). Advances in technology and the declining costs of DNA sequencing have spurred global efforts to discover differentially expressed genes in various cancers. From one such effort, TC2N was found to be widely upregulated in several human cancers, including lung, breast, and gastric cancers. TC2N levels were correlated with multiple clinicopathological features and prognosis, such as TNM stage, histological grade, tumor size, overall survival, lymph node metastasis, and distant metastasis. In support of its involvement in tumorigenesis and tumor progression, *in vitro and in vivo* experiments have shown that TC2N affects proliferation, apoptosis, migration, invasion of tumor cells and tumor growth in many cancers. The underlying molecular mechanisms of TC2N in several cancers have also been preliminarily explored and suggest that TC2N modulates several key signaling pathways that influence carcinogenesis and cancer progression, including p53, NF-κB, and PI3K/AKT signaling pathways.

Although TC2N is a potential therapeutic target, several questions remain to be addressed. First, the molecular mechanism of TC2N in different types of cancers is not completely understood. For example, while preliminary data suggest that in gastric cancer TC2N modulates the expression of several cancer related genes, the specific pathway affected by TC2N is unclear. Furthermore, while the function of TC2N in lung, breast and gastric cancer have been studied to some extent, its potential role in other cancers, such as cancers associated with the urinary and reproductive systems remain unexplored. Second, while TC2N is upregulated in tumor tissues of some specific cancers, it is not known if TC2N is also upregulated in body fluids such as plasma and urine. The identification of diagnostic biomarkers is a promising avenue for early cancer diagnosis. If TC2N is detectable in plasma or urine, it may facilitate early detection and prognosis assessment using simple and non-invasive tests. Third, it is unknown if TC2N is a tumor-associated antigen and requires further evaluation. Fourth, TC2N is an immune system associated gene, but whether it can serve as a target in personalized immunotherapies remains to be seen. Therefore, more attention should be paid to the clinical value of TC2N in cancer diagnosis and treatment.

In summary, TC2N has been shown to have oncogenic or tumor-suppressive functions in different types of cancers, and could potentially serve as a cancer-specific molecular biomarker for early diagnosis, treatment, and prognosis assessment. While some progress has been made in the mechanistic analysis of TC2N, several questions remain unanswered. Future work needs to focus on understanding the precise molecular mechanism of TC2N in carcinogenesis and tumor progression to explore the potential clinical application of TC2N.

## Author Contributions

HL and TW contributed to the conception and design of the review. HL wrote the first draft of the manuscript. BL revised the manuscript. All authors contributed to the article and approved the submitted version

## Funding

This work was supported by National Key R&D Program of China (Grant #2018YFC1311600), Jilin Province Financial and Health Project (Research on the Differential Expression of Cyclic RNA in Cervical Cancer HeLa Cells and the Mechanism of Radiation Resistance under Radiation Induction); Jilin Provincial Department of Finance (Consortium of Medical Consortium for Diagnosis and Treatment of Difficult Women’s Tumors and Precision Radiotherapy Training Base Construction Project); Jilin Province Medical and Health Talent Special Project (2019SCZT010); National Key Clinical Specialty Capacity Building Project (Application of Non-coplanar 3D Printing and Intertissue Interpolation Technology in Improving the Diagnosis and Treatment Ability of Recurrent and Refractory Cervical Cancer).

## Conflict of Interest

The authors declare that the research was conducted in the absence of any commercial or financial relationships that could be construed as a potential conflict of interest.

## Publisher’s Note

All claims expressed in this article are solely those of the authors and do not necessarily represent those of their affiliated organizations, or those of the publisher, the editors and the reviewers. Any product that may be evaluated in this article, or claim that may be made by its manufacturer, is not guaranteed or endorsed by the publisher.

## Abbreviations

TC2N, tandem C2 domains nuclear protein; TAAs, tumor-associated antigens; CDK5, cyclin-dependent kinase 5; P21(CDKN1A) , cyclin dependent kinase inhibitor 1A; P53, tumor protein p53; BAX, a member of the B-cell lymphoma-2(BCL2) gene family; BCL1, B-cell lymphoma-1; IκB, inhibitor of NF-κB; NF-κB, nuclear factor kappa-light-chain-enhancer of activated B cells; MMP7, matrix metalloproteinase 7; MMP9, matrix metalloproteinase 9; ALK, anaplastic lymphoma kinase; EBP1, ErbB-3 binding protein 1; AKT, serine threonine kinase; GSK3β, glycogen synthase kinase-3β; PTEN, phosphatase and tensin homolog deleted on Chromosome 10; MMP2, matrix metalloproteinase 2; GALNT3, polypeptide N-acetylgalactosaminyltransferase 3; RBM47, RNA binding motif protein 47.
